# The stromal immunoexpression of CLIC4 may be related to the difference in the biological behavior between oral squamous cell carcinoma and oral verrucous carcinoma

**DOI:** 10.4317/medoral.25842

**Published:** 2023-04-07

**Authors:** Mariana Carvalho Xerez, Caio César da Silva Barros, Salomão Israel Monteiro Lourenço Queiroz, Éricka Janine Dantas da Silveira, Antonio de Lisboa Lopes Costa

**Affiliations:** 1 DDS, MSc, Ph.D. student, Postgraduate Program in Dental Sciences, Department of Dentistry, Federal University of Rio Grande do Norte, Natal, RN, Brazil; 2 DDS, MSc, Ph.D., Postgraduate Program in Oral Pathology, Federal University of Rio Grande do Norte, Natal, RN, Brazil; 3 DDS, MSc, Ph.D., Professor, Postgraduate Program in Dental Sciences, Department of Dentistry, Federal University of Rio Grande do Norte, Natal, RN, Brazil

## Abstract

**Background:**

Oral squamous cell carcinoma (OSCC) has high morbidity and mortality rates while oral verrucous carcinoma (OVC), an uncommon variant of OSCC, exhibits a distinct biological behavior. CLIC4 protein plays a role in the cell cycle and apoptosis regulation and participates in the myofibroblasts transdifferentiation process, which are the main cells of the tumor stroma. This study analyzed the immunoexpression of CLIC4 and α-SMA in 20 OSCC cases and 15 OVC cases.

**Material and Methods:**

A semiquantitative analysis of CLIC4 and α-SMA immunoexpression was performed in the parenchyma and stroma. Nuclear and cytoplasmic reactivity was analyzed separately for the CLIC4 immunostaining. The data were submitted to Pearson's chi-square and Spearman's correlation tests (*p* ≤ 0.05).

**Results:**

In the CLIC4 analysis, there was a significant difference in the immunoexpression of this protein between OSCC and OVC stroma (*p* < 0.001). It was observed a higher expression of α-SMA in the OSCC stroma. There was a positive and significant correlation between CLIC4 and α-SMA immunoexpression in the OVC stroma (r = 0,612; *p* = 0,015).

**Conclusions:**

The decrease or absence of nuclear CLIC4 immunoexpression in the neoplastic epithelial cells and the increase of its expression in the stroma may influence the difference in biological behavior between OSCC and OVC.

** Key words:**Squamous cell carcinoma, verrucous carcinoma, CLIC4, myofibroblasts.

## Introduction

Oral carcinogenesis is a multiphase complex process that involves the interrelation of factors capable of promoting genetic alterations in oral keratinocytes, leading them to a malignant phenotype (1,2). Among the malignant neoplasms that affect the oral cavity, oral squamous cell carcinoma (OSCC) is the most frequent and has high mortality rates. On the other hand, oral verrucous carcinoma (OVC) is an uncommon and distinct variant of OSCC, which exhibit specific morphology and biological behavior (3-6).

Intracellular chloride channels (CLIC) are proteins whose functions are related to cell cycle regulation. Among them, CLIC4 stands out since it is involved in the pathogenesis of several malignant neoplasms (7,8). CLIC4 varies its expression pattern in normal tissue cells, in which it may be present both in the plasma membrane and in various intracellular organelles, such as the mitochondrial membrane. However, it is observed that in the parenchyma of malignant neoplasms, this protein shows high cytoplasmic expression and is related to several changes in cell behavior. On the other hand, in the tumor stroma, its downregulation acts in the process to change the mesenchymal cell phenotype from fibroblast to myofibroblast (9-12).

Cancer-associated fibroblasts (CAF), also called myofibroblasts, are the main tumor stroma cellular constituents of many carcinomas. These cells present a hybrid phenotype with characteristics of fibroblasts and smooth muscle cells, thus expressing the α isoform of smooth muscle actin (α-SMA) (13,14). Studies show that CAF presence in the tumor stroma stimulates the neoplastic cells' proliferation and, through the synthesis and secretion of extracellular matrix (ECM) proteins and proteases, creates an environment permissive to the tumor invasion and metastasis process (13,15). In this context, given the functions of CLIC4 and the participation of CAF in promoting tumor growth and invasion, the present study analyzed the immunoexpression of CLIC4 and α-SMA in OSCC and OVC since these malignant neoplasms exhibit different biological behavior.

## Material and Methods

- Study design and sample

In this retrospective cross-sectional study, it was analyzed stored tissue blocks of 20 cases of OSCC and 15 cases of OVC diagnosed at the Oral Pathology Service of the Federal University of Rio Grande do Norte (Natal, RN). All selected OSCC and OVC cases were morphologically reassessed through routine staining (hematoxylin and eosin). Clinical information regarding gender, age, anatomical location, and histopathological diagnosis were collected from the patients' biopsy records retrieved in the service aforementioned. It was included cases with a histopathological diagnosis of OSCC and CVO located in the oral mucosa, while it was excluded cases with insufficient amount of biological material to perform the immunohistochemical study unfeasibly.

- Immunohistochemical staining

For immunohistochemical analysis, 3-µm thick tissue sections were mounted on organosilane-coated slides (3-aminopropyltriethoxysilane; Sigma Chemical Co., St. Louis, MO, USA). Deparaffinization, rehydration, and antigen retrieval were performed using Trilogy (Cell Marque, CA, USA) diluted in distilled water (1:100) and heated in a Pascal pressure cooker. Endogenous peroxidase and nonspecific antibody reactions were blocked with 3% hydrogen peroxide and Protein Block (Thermo Scientific, Runcorn, UK). The tissues were incubated with primary antibodies anti-CLIC4 (EPR14253, Abcam, 1:4000, 60’) and anti-α-SMA (1A4, DAKO, 1:800, 60’). Antibodies were detected using the HiDef DetectionTM HRP Polymer system (Cell Marque) and the reaction was developed with diaminobenzidine as chromogen (DAB, Sigma Chemical, St Louis, MO, USA). The sections were counterstained with Mayer’s hematoxylin and mounted in Permount® (Fisher Scientific, Fair Lawn, NJ, USA). Replacement of the primary antibodies with bovine serum albumin was used as the negative control and human melanoma tissue served as the positive control.

- Immunostaining assessment

All immunohistochemical slides were scanned with a digital slide scanner system (3DHISTECH®, Budapest, Hungary), and further analyzed by one previously trained examiner in the Pannoramic Viewer 1.15.2 software (3DHISTECH®, Budapest, Hungary). CLIC4 immunoexpression was evaluated semiquantitatively in the parenchyma and stroma, while α-SMA immunoexpression was evaluated semiquantitatively in the stroma. It was considered positive cells for CLIC4 and α-SMA immunostaining the cell with brown staining in the nucleus or cytoplasm.

CLIC4 analysis in the tumor epithelial cells was performed as described by Piva *et al*. (16) according to the scores: 1 (negative /low expression; < 5% of positive cells), score 2 (moderate expression; 5 - 50% positive cells), or score 3 (high expression; > 50% positive cells). On the other hand, in the tumor stroma, the cells of fusiform morphology positive for CLIC4 and α-SMA were categorized as proposed by Paral *et al*. (17): score 0 (negative); score 1 (focal positivity; < 50% positive cells); score 2 (strong positivity; > 50% positive cells). The endothelial cells were excluded from the tumor stroma analysis.

- Statistical analysis

Data were analyzed using the IBM Statistical Package for the Social Sciences (SPSS 22.0; IBM Corp., Armonk, United States of America). Descriptive statistics were used for the characterization of the sample. The weighted kappa coefficient was calculated to assess the intraobserver agreement in the immunohistochemical analysis (≤ 0.20, slight agreement; 0.21 to 0.40, fair agreement; 0.41 to 0.60, moderate agreement; 0.61 to 0.80, good agreement; 0.81 to 1, excellent agreement). In this context, the intraobserver agreement was excellent (weighted kappa coefficient 0.82). Associations between nominal variables were analyzed using Pearson's chi-squared test. Spearman’s correlation coefficient was used to verify possible correlations between the CLIC4 and α-SMA immunoexpression in the stroma of OSCC and OVC. The level of significance was set at 5% (*p* ≤ 0.05) for all tests.

## Results

- Clinicopathological data

Demographic and clinicopathological characteristics are presented in Table 1. OSCC was more frequent in male patients (n = 11; 55%) with a mean age of 66.9 ± 16.3 years old, and the tongue was the most affected anatomical location (n = 9; 45%). On the other hand, OVC was more frequent in female patients (n = 9; 60%) with a mean age of 61.7 ± 12.7 years old. The inner mucosa of the lip (n = 4; 26.6%) was the most affected anatomical location and the alveolar ridge was affected in four cases.

- CLIC4 immunoexpression in OSCC and OVC

**Figure 1:**
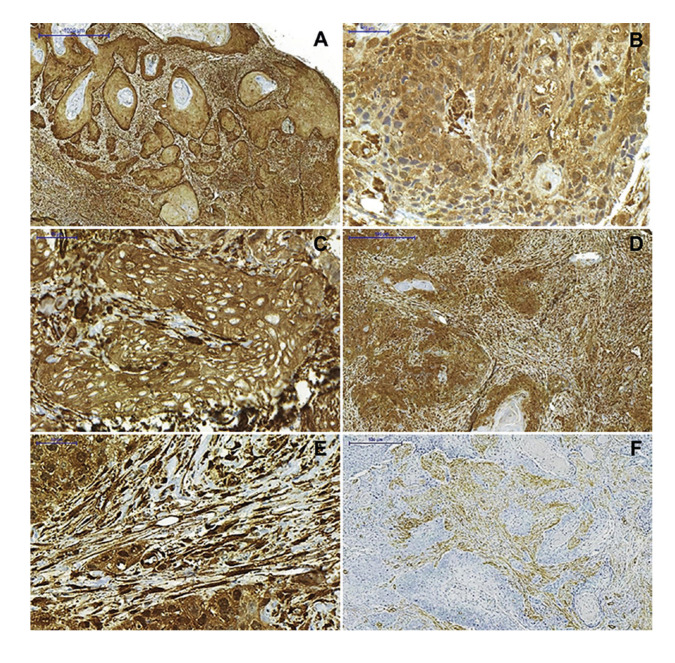
Immunoexpression of CLIC4 and α-SMA in OSCC. (A) CLIC4 immunoexpression in the parenchyma was absent in the keratin pearls. (B) Positive immunoexpression of CLIC4 in the nucleus and cytoplasm in neoplastic epithelial cells. (C) Absence of nuclear immunostaining in neoplastic epithelial cells. (D and E) CLIC4 and (F) α-SMA immunoexpression in spindle cells of tumor stroma [Scale bars: 1000 µm (A), 500 µm (D and F), and 50 µm (B, C, and E)].

**Figure 2:**
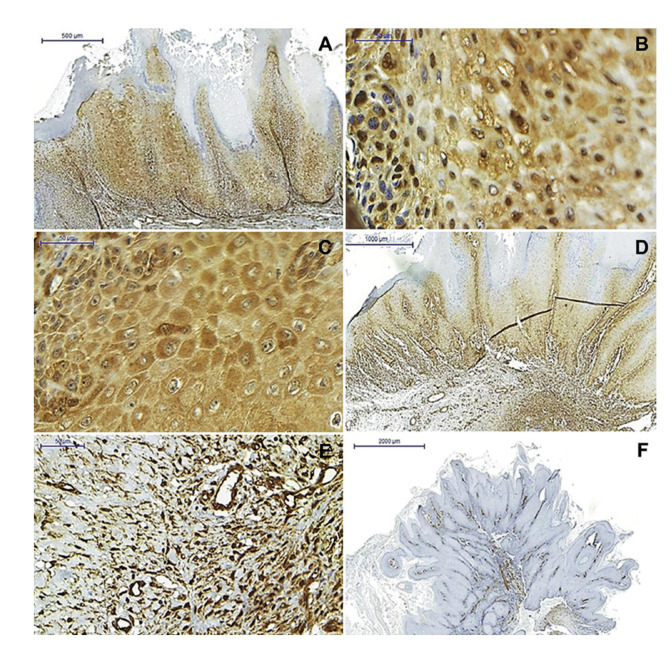
Immunoexpression of CLIC4 and α-SMA in OVC. (A) CLIC4 immunoexpression in the parenchyma of the tumor. (B) Nuclear and cytoplasmatic immunostaining of the neoplastic epithelial cells. (C) Cytoplasmic immunostaining in the neoplastic epithelial cells. (D and E) CLIC4 and (F) α-SMA immunoexpression in spindle cells of tumor stroma [Scale bars: 2000 µm (F), 1000 µm (D), 500 µm (A), and 50 µm (B, C, and E)].

CLIC4 immunoexpression in OSCC and OVC was heterogeneous in neoplastic epithelial cells and a predominantly cytoplasmic immunostaining pattern was observed, as well as simultaneous immunostaining of the nucleus and cytoplasm (Fig. 1, Fig. 2). There was not exclusively nuclear immunoexpression in any of the analyzed cases (Fig. 1, Fig. 2). CLIC4 immunoexpression in the OSCC parenchyma was observed in all the extension of the tumor, except in the areas where keratin pearls were formed (Fig. 1). Similarly, its immunoexpression in OVC parenchyma was observed throughout the entire extension of the neoplastic epithelium, except in the stratum corneum (Fig. 2). No statistically significant result was observed when comparing nuclear, cytoplasmic, and total immunostaining (nucleus and cytoplasm) in the neoplastic epithelial cell of OSCC and OVC (Table 2).

The analysis of CLIC4 immunoexpression in the tumor stroma showed high expression of this protein in OSCC when compared to OVC (Fig. 1, Fig. 2). In addition, a significant difference (*p* < 0.001) was observed when comparing CLIC4 immunoexpression between the two neoplasms, in which OSCC cases were 6.76 times more frequent (95% confidence interval: 2.33 - 19. 60) of score 2 (Fig. 1) compared to OVC cases (Fig. 2) (Table 2).

- α-SMA immunoexpression in tumor stroma

The analysis of α-SMA immunostaining in mesenchymal cells revealed a higher frequency of score 2 in cases diagnosed as OSCC (n = 14; 70%) (Fig. 1), while there was a higher frequency of score 1 in cases of OVC (n = 9; 60%) (Fig. 2). However, no statistically significant difference was observed in α-SMA immunoexpression between the two neoplasms.

**Table 1:**
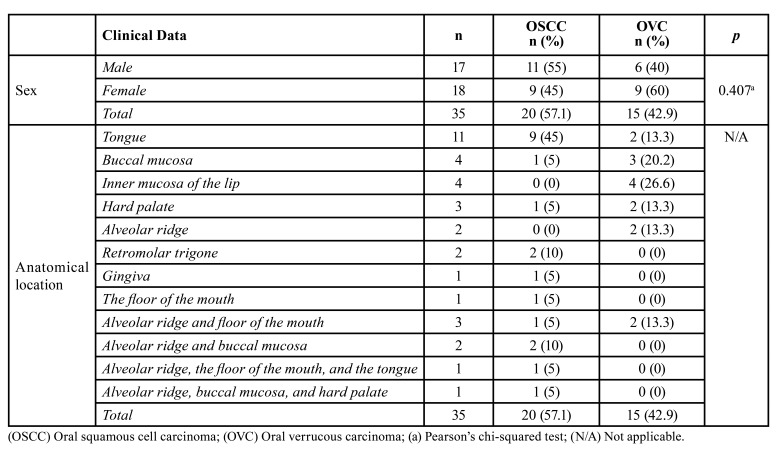
Sample size, absolute and relative frequency of oral squamous cell carcinoma and oral verrucous carcinoma according to the sex and anatomical location.

**Table 2:**
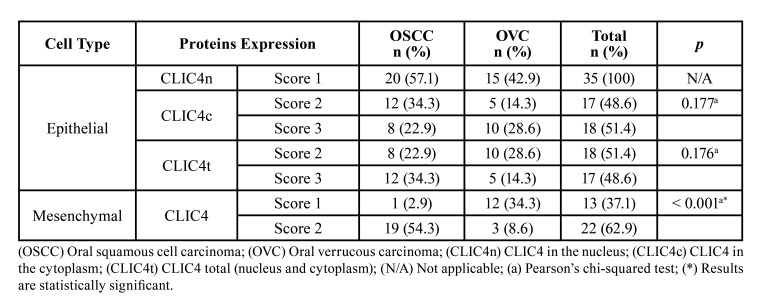
CLIC4 immunoexpression in neoplastic epithelial and mesenchymal cells in oral squamous cell carcinoma and oral verrucous carcinoma.

- Correlation of proteins immunoexpression in the tumor stroma

Correlation analysis in the stroma revealed a positive and not significant correlation between CLIC4 and α-SMA immunoexpression in OSCC cases (r = 0.350; *p* = 0.130), and a positive and statistically significant correlation of these proteins in OVC cases (r = 0.612; *p* = 0.015).

## Discussion

OVC is an uncommon variant of OSCC that exhibits distinct clinical and histopathological features. The literature demonstrates differences in the clinical aggressiveness between these two neoplasms. In this way, unlike OSCC, OVC presents itself as a well-differentiated, slow-growing, exophytic, and locally invasive lesion with little tendency to metastasize (7,18). To the best of our knowledge, this is the first study that investigated the CLIC4 and α-SMA immunoexpression in OSCC and OVC. Our results showed that CLIC4 may participate in the CAF differentiation, which may reflect the differences in biological behavior between these neoplasms.

Oral carcinogenesis is a complex process in which the tumor microenvironment plays a critical role in creating a permissive environment for the invasion and metastasis process of neoplastic cells (19,20). In this way, understanding the molecular modifications that occur in tumor cells can provide important information for predicting the biological behavior of malignant neoplasms. CLIC4 is an ion channel that participates in the physiological activity and homeostasis of the cells by performing functions related to fluid and ionic transport as well as intracellular pH maintenance. In normal epithelial cells, CLIC4 is present in the cytoplasm and mitochondrial membrane and exhibits high nuclear expression, and its functions are related to metabolic stress, inhibition of cell proliferation, and apoptosis (7-10,21).

On the other hand, CLIC4 expression in neoplastic cells is elevated at the cytoplasmic level and downregulated or absent in the nucleus, thus demonstrating its participation in carcinogenesis (7,8,10). In a recent study, Lima *et al*. (21) analyzed CLIC4 immunoexpression in lower lip squamous cell carcinoma (LLSCC) and actinic cheilitis (AC). These authors observed a decrease in the nuclear CLIC4 immunoexpression and an increase in its cytoplasmic immunoexpression in the neoplastic cells of the CCELI when compared to the epithelial cells of the AC with a high and low risk of malignant transformation. Thus, these authors suggested that the change in the CLIC4 immunoexpression pattern indicates its participation in lip carcinogenesis.

In our study, CLIC4 immunoexpression was evaluated in two epithelial neoplasms with different biological behavior. It was observed that, in both OSCC and OVC, CLIC4 exhibited cytoplasmic immunostaining or simultaneous staining in the nucleus and cytoplasm of neoplastic epithelial cells, and it was not evidenced exclusively nuclear expression in any of the lesions. In addition, no significant difference was evidenced when comparing the CLIC4 immunostaining between OSCC and OVC neoplastic epithelial cells. The literature reports that cellular redox status plays a critical role in CLIC4 conformation regulation and nuclear translocation. Also, it is known that malignant tumor cells are in a potential state of oxidative stress and produce high levels of reactive oxygen species, which are involved in the initiation and progression of carcinogenesis (9,22-25). In this way, we believe that the absence of exclusively nuclear immunostaining can be due to the production of antioxidant molecules, such as glutathione, which can inhibit the CLIC4 modifications necessary for nuclear translocation, a fact that prevents its action in controlling cell proliferation and apoptosis (7,22).

In addition, the literature demonstrates that the immunoexpression of other proteins denotes the similarity of these lesions regarding cell proliferation and apoptosis. In this context, Angadi and Krishnapillai (26) analyzed cyclin D1 immunoexpression in OSCC and OVC neoplastic cells. It is known that cyclin D1 plays a role in regulating the progression from the G1 to S phase during the cell cycle and that it is associated with reduced expression of anti-apoptotic proteins, such as Bcl-2 (26). These authors observed that there was no difference in the expression of this protein between the two neoplasms, thus denoting its importance in the proliferative autonomy of the neoplastic epithelial cells of both lesions (26).

Concerning the CLIC4 role in the tumor stroma, we observed a significantly higher immunoexpression in the OSCC stroma. This finding may be related to the expression of transforming growth factor beta (TGF-β), which is related to the stromal cells differentiation process that constitutes the tumor microenvironment (10,12,21). The principal stromal cells are the CAF, which is highly specialized and differs from normal fibroblasts by α-SMA higher expression and by the ability to synthesize EMC proteins, proteinases, and growth factors in high levels (13,15,27-29).

Our study evidenced a higher α-SMA expression in the OSCC stroma (Score 2) compared to OVC cases (Score 1). Although these findings were not significant, they corroborate the results of Chaudhary *et al*. (30), that when investigating the α-SMA immunoexpression, observed its significantly higher expression in the OSCC stroma when compared to the OVC. In this way, these authors highlighted that this high immunoexpression could be associated with tumors that exhibit a more invasive phenotype. Furthermore, our results revealed a positive and significant correlation between CLIC4 and α-SMA immunoexpression in CAF of the OVC. It is important to emphasize that CLIC4 and α-SMA are upregulated proteins co-located in the myofibroblasts present in the tumor stroma, which can demonstrate CLIC4 participation in the differentiation process of these cells (10,12,21). Thus, we believe that the low CLIC4 and α-SMA immunoexpression and the significant correlation between their expression can be related to the OVC’s lower invasiveness capacity compared to oral squamous cell carcinoma.

In this context, the literature shows that CLIC4 is positively regulated by TGF-β, which also regulates mechanisms related to cell growth, differentiation, apoptosis, and adhesion. In the TGF-β pathway, these processes are regulated by preventing the dephosphorylation of phospho-Smad2 and 3 in the nucleus. It has been reported that several proteins promote the protection of phospho-Smads from the action of phosphatases, and CLIC4 is the main protein with this protective function (10-12,21). Also, it is known that CLIC4 is the most regulated gene during the differentiation of myofibroblasts via the TGF-β pathway, which is related to increased α-SMA expression (10-12,21).

We emphasize that in our study, CLIC4 and α-SMA analysis in OVC was performed in the subepithelial region, so the evaluated stromal cells were in proximity to the neoplastic cells. Otherwise, incisional biopsies were used in OSCC cases due to the difficulty in obtaining the surgical specimens for the evaluation of the tumor invasion front, which constitutes a limitation of our study. Despite this, our results demonstrate that the absence or reduction of CLIC4 nuclear immunostaining in neoplastic epithelial cells may constitute an event related to the loss of control in the mechanisms associated with proliferation and apoptosis in these cells. In addition, the decrease of CLIC4 immunoexpression in stromal myofibroblasts influences the α-SMA expression in OVC, suggesting that the CLIC4 expression may be related to the differences in the biological behavior of OSCC compared to oral verrucous carcinoma.
